# Electromyography-Based Quantitative Representation Method for Upper-Limb Elbow Joint Angle in Sagittal Plane

**DOI:** 10.1007/s40846-015-0033-8

**Published:** 2015-04-25

**Authors:** Muye Pang, Shuxiang Guo, Qiang Huang, Hidenori Ishihara, Hideyuki Hirata

**Affiliations:** Graduate School of Engineering, Kagawa University, Takamatsu, 761-0396 Japan; Department of Intelligent Mechanical Systems Engineering, Kagawa University, Takamatsu, 761-0396 Japan; School of Life Science and Technology, Beijing Institute of Technology, Beijing, 100081 China

**Keywords:** Upper limb elbow joint, Electromyography (EMG), Hill-type model, State switching, Continuous representation

## Abstract

This paper presents a quantitative representation method for the upper-limb elbow joint angle using only electromyography (EMG) signals for continuous elbow joint voluntary flexion and extension in the sagittal plane. The dynamics relation between the musculotendon force exerted by the biceps brachii muscle and the elbow joint angle is developed for a modified musculoskeletal model. Based on the dynamics model, a quadratic-like quantitative relationship between EMG signals and the elbow joint angle is built using a Hill-type-based muscular model. Furthermore, a state switching model is designed to stabilize the transition of EMG signals between different muscle contraction motions during the whole movement. To evaluate the efficiency of the method, ten subjects performed continuous experiments during a 4-day period and five of them performed a subsequent consecutive stepping test. The results were calculated in real-time and used as control reference to drive an exoskeleton device bilaterally. The experimental results indicate that the proposed method can provide suitable prediction results with root-mean-square (RMS) errors of below 10° in continuous motion and RMS errors of below 10° in stepping motion with 20° and 30° increments. It is also easier to calibrate and implement.

## Introduction

Electromyography (EMG) has been applied to various fields. Fukuda et al. [1] used EMG signals to control a manipulator. They adopted a statistical neural network, named the log-linearized Gaussian mixture network, to achieve robust discrimination against differences among individuals, electrodes locations, and time variations caused by fatigue or sweat. They reported that the method can provide smooth control for the manipulator and it might allow a physically handicapped person to sense a feeling of prosthetic control similar to that of the original limb. Liarokapis et al. [2] used EMG signals from sixteen muscles of the upper limb to study the muscular co-activation patterns during a variety of reach-to-grasp motions. EMG signals reflect the level of muscle activation, and can thus be used to predict or recognize human motion [3–7]. This kind of technology is especially useful for the physically handicapped person, as applied by Fukuda [1]. EMG signals can be used for intuitive control. Artemiadis et al. [8] developed a switching regime model to decode the EMG activity from 11 muscles into a continuous representation of arm motion in three-dimensional space. They reported that this switching regime model can overcome some main difficulties of EMG-based control systems, such as the nonlinearity of the relationship between the EMG recordings and the arm motion, as well as the non-stationarity of EMG signals with respect to time. A Bayesian classifier was applied for each muscle to compensate for the various features of EMG signals. Besides motion interpretation, EMG signals can be used to represent human arm stiffness [9, 10]. Ajoudani et al. [11] used EMG signals from eight muscles around the operator’s arm to derive stiffness information, which was sent with the position command to a slave robot to achieve tele-impedance control.

Despite progress, the non-stationarity of EMG signals and the uncertainty of how the central nervous system controls human motion make it difficult to implement EMG control outside a laboratory environment. Pattern recognition methods have been used by researchers to map EMG signals to the target behavior. Chen et al. [12] developed a multi-kernel learning support vector machine method to classify multiple finger movements. In order to recognize hand motions, Tang et al. [13] applied a multi-channel energy ratio feature extraction method to overcome the influence of various forces for a given gesture. They used the proposed feature extraction method and a cascaded-structured classifier to recognize eleven hand gestures. Phinyomark et al. [14] implemented twelve anthropometric variables to design an automatic/semi-automatic calibration system for EMG recognition. Although many works have been done and high classification accuracy has been achieved [15–17], one of the disadvantages of pattern recognition methods is non-smooth control. The results which are usually used as reference commands are discrete for controller, where continuous results are desired. Moreover, many electrodes are needed to record muscle’ activation behaviors to improve recognition results.

Some physiological models have been used to estimate musculotendon forces using EMG signals, such as Huxley- [18, 19] and Hill-type models [20]. For musculotendon force calculation, EMG signals are used to represent the muscle activation level, which is used with other parameters in the musculoskeletal model to obtain the desired force value. Compared with the complexity of Huxley-type models, Hill-type models are more computationally viable. Cavallaro et al. [21] developed a Hill-type-model-based myoprocessor to predict joint torque. Seven muscles around the upper limb were recorded and a genetic algorithm was implemented to tune the parameters of the model. Manal et al. [22] used a Hill-typed model to calculate muscle force and implemented a forward dynamics approach to estimate joint angle. They used an optimal controller to map the relationship between measured and predicted joint moments. Although using the forward muscular model to calculate human body motions is attractive, there are some difficult problems that prevent its implementation. One of the problems is the redundancy of human muscles around joints. It is almost impossible to get each muscle’s status around the joint. Another problem is the parameters involved in the Hill-type model. In addition to the muscle activation level, the muscle length changes, muscle velocity changes, and pennation angle are usually used in the model. Accurately estimating these parameters is difficult. A musculoskeletal model can be used to build a quantitative relation between EMG signals and musculotendon forces.

In this paper, a continuous upper limb elbow joint angle representation method is proposed. Single-channel EMG signals recorded from the biceps muscle are used as the input of the proposed method. A quantitative relationship between EMG signals and joint angles is developed using a Hill-type musculoskeletal model. Because only EMG signals are used, some parameters involved in the musculoskeletal model can not be evaluate by measurement and thus introduce error. Therefore, a state switching model is developed to avoid the influence of these factors. The tested movement of the upper limb is voluntary elbow flexion and extension in the sagittal plane. The involved movement includes concentric contraction motion (elbow flexion), isometric contraction motion (elbow holding), and active shortening motion (elbow extension). Using the proposed method, elbow joints can be represented only with EMG signals in these three types of contraction motion. The results are used as reference control commands for a developed upper limb exoskeleton device (ULED). To estimate the efficiency of the proposed method, a continuous test and a consecutive stepping test were performed.

## Materials and Methods

### Experimental Setup

Ten healthy volunteers (age: 24.60 ± 1.67 years, height: 1.70 ± 0.07 m, weight: 67.66 ± 9.54 kg, two female, eight male, two left-handed, and eight right-handed) participated in the experiments. Before placing the electrode, which was aligned parallel to the muscle fibers, over the belly of the muscle, the skin was shaved and cleaned with alcohol in order to reduce skin impedance. The sampling rate was 1000 Hz with differential amplification (gain: 1000) and common mode rejection (104 dB). A fourth-order high-pass Butterworth filter with a 10-Hz cut-off frequency was implemented in software to remove the DC offsets in EMG signals before they were rectified. The user interface was programmed using Visual C++ 2010 (Microsoft Co., USA). The analog/digital (A/D) data from the A/D board was collected through the application programming interface and processed with MATLAB (The MathWorks Co., USA). The software was run on a personal computer with a 2.8-GHz quad-core processor (Intel Core i7 860) and 4 GB of RAM. An MTx sensor (Xsens Technologies B.V., USA) was attached on the subject’s forearm to record the elbow joint angle for calibration and comparison.

**Fig. 1:**
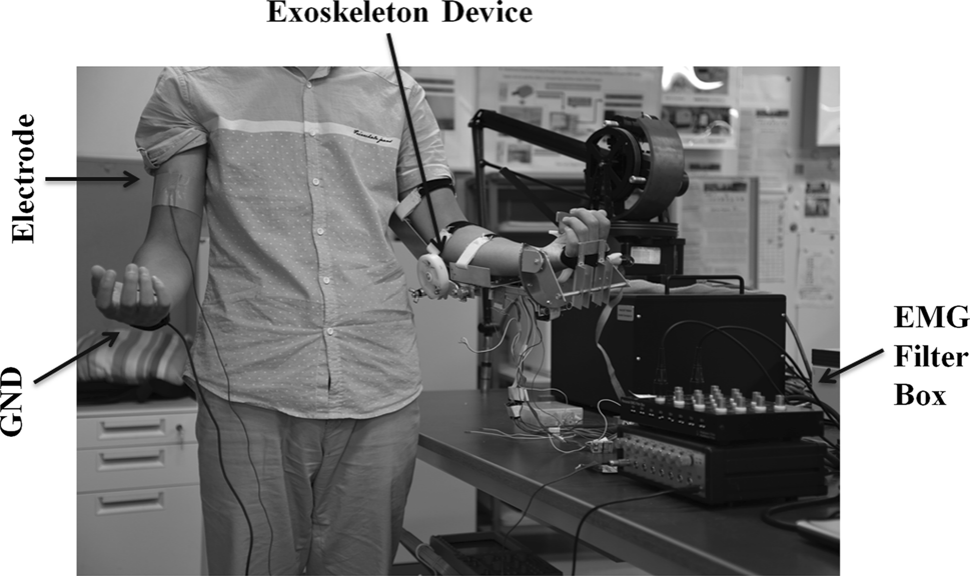
Experimental setup. The electrode is attached on one of the subject’s *upper arm* (on the surface of the biceps brachii) and the ULED is placed on the other arm. The ULED is driven by the prediction results using the proposed method

The proposed method was implemented to control the developed ULED to estimate its efficiency. The ULED was designed to provide passive and active rehabilitation training for stroke patients. There are three active degrees of freedom (DoFs) (one for the elbow joint and tow for the wrist joint) and four passive DoFs (two for the elbow joint and two for the wrist joint). The total weight of this device is 1.3 kg, making it suitable for home rehabilitation. The details of the ULED can be found elsewhere [23]. The proposed method can be used for home bilateral rehabilitation with the ULED. During home bilateral rehabilitation training, the hemiparesis patient can wear the ULED on the impaired upper limb and the proposed method can control the ULED using EMG signals recorded from their intact upper limb. Because the EMG signals are recorded from the intact upper limb, the healthy volunteers can take place of the patients in the experiments. The experimental setup is shown in Fig. 1.

### Experimental Protocol

A maximum voluntary contraction (MVC) test at the isometric contraction condition was performed before the experiment. Subjects were asked to hold a dumbbell (from 3 to 18 kg) at an angle of 90° between their upper arms and forearms and the EMG signals from the biceps muscle were recorded. Five trials were performed to determine the MVC EMG value. A sufficient rest time between the five trials was provided to avoid muscle fatigue.

In the experiment of upper limb flexion and extension in the sagittal plane, the subjects were asked to start with both side of their arms (one side, namely active arm, is used to record EMG signals, and the other side, namely passive arm, is with ULED worn on) relaxed vertically and then flex their forearms to 90°. After having maintained their forearms in the horizontal position for 3 s, the subjects were asked to extend their forearms to the initial vertical position. Then, a calibration calculation for proposed method was performed offline. After the calibration was finished, the subjects were asked to wear the ULED and perform online experiments. The subjects moved their active arms with the motion used in the previous experiment and the ULED carried the other arms to move passively.

Furthermore, a consecutive stepping test was performed by five of the ten subjects. In the consecutive stepping test, the subjects were asked to move their active upper limb to angle of 30°, 20°, and 10°, respectively, and hold for 3 s at each step.

A photograph of each subject was taken to record the electrode position on the upper arm. The condition of upper limb movement, such as forearm rotation speed and upper arm stiffness, should be restricted for the accurate offline calibration. In order to keep the rotation speed and generalize upper limb movement, the subjects were asked to practice the motion by following a prerecorded video. All motions were voluntary without any external force applied on the upper limb. Each subject repeated the three experiments ten times with a relaxation time of 1 min between tests.

### Musculoskeletal Model

**Fig. 2:**
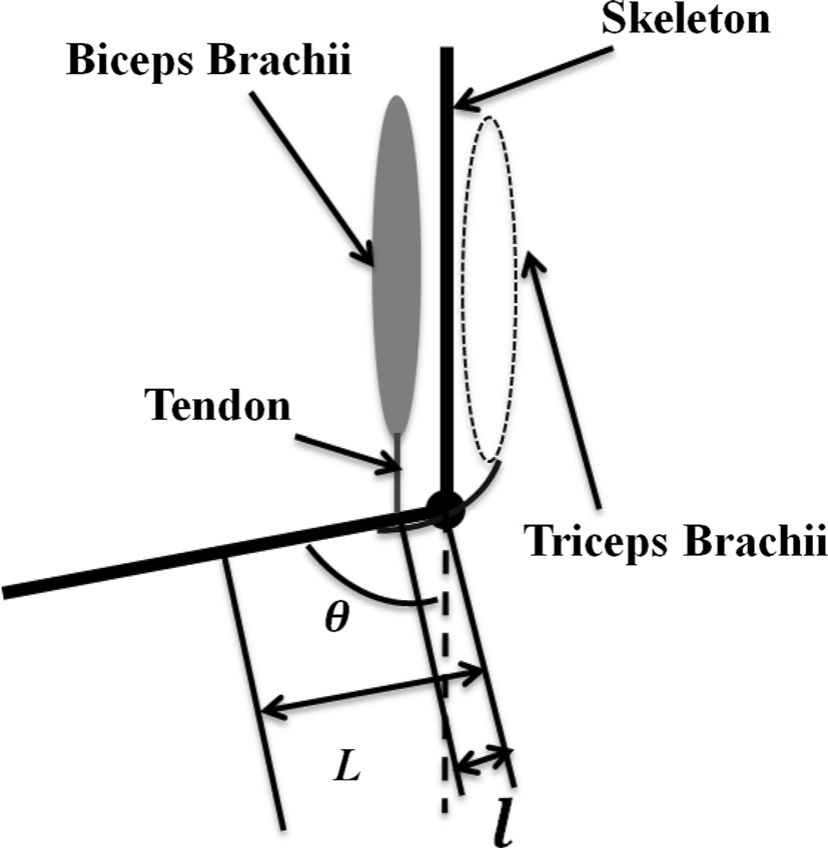
Side view of proposed muscular skeleton model in *vertical plane*

A side view (in the sagittal plane) of the proposed musculoskeletal model is shown in Fig. 2. The distance between the attachment point of the tendon to the skeleton and elbow joint is *l*. According to a previous study [24], the tendon in the upper arm can be regarded as having high stiffness and thus the tendon deformation is zero. The deformation of the muscle–tendon that results in elbow rotation can thus be regarded as resulting only from muscle contraction. The elbow angle *θ* is the one to be predicted. *L* is the distance between the forearm centroid and the elbow joint. In the sagittal plane, it can be considered that the biceps muscle contracts to pull the forearm against the force of gravity during the motion of elbow flexion and extension and that the triceps muscle remains almost unactivated. No obvious EMG signal changes from the triceps muscle can be observed during elbow flexion and extension in the sagittal plane. In the transverse plane, the triceps has to pull the forearm to extend the elbow. As only voluntary motion in the sagittal plane is discussed, the effect from triceps brachii is ignored in this paper.

The following equation describes the motion of elbow flexion and extension in the sagittal plane:

1 $$F_{B} l\,\sin \,\theta - mgL\,\sin \,\theta - \tau_{f} = I\ddot{\theta } + \tau_{e}$$

where *F*_*B*_ is the musclotendon force exerted by the biceps and τ_*f*_ represents the torque from frictional effects, which is assumed to be zero in this paper. τ_*e*_ represents the effects of the environmental interaction, which is also assumed to be zero because the subjects held nothing in their hands when they performed the experiments. The mass and inertia of the forearm are *m* and *I*, respectively. Dividing Eq. (1) by *lsinθ* on both sides yields:

2 $$F_{B} = mgLl^{ - 1} + Il^{ - 1} \ddot{\theta }\,\sin \,\theta$$

For Eq. (2), it is difficult to accurately estimate *m* and *I*. However, these two parameters can be assumed to be constant during a certain period of time. Using a Hill-type muscular model, the term *F*_*B*_ can be represented as a function of muscular activation level (*a*), muscle contraction length (*L*_*m*_), and muscle contraction velocity (*dL*_*m*_). Equation (2) can thus be rewritten as:

3 $$f(a,L_{m} ,dL_{m} ) = c_{1} + A_{1} \ddot{\theta }\,\sin \,\theta$$

where *c*_*1*_ equals *mgLl*^−1^ and *A*_*1*_ equals *Il*^−1^.

An actual musculoskeletal model of human upper limb is more complex than the proposed one, for example not only biceps brachii is involved in the elbow flexion but also brachialis muscle. The brachialis is the deep muscle in the upper arm. It is not easy to record EMG signals from the brachialis using non-invasive surface electrodes. It is assumed that the muscle synergies involved in a certain motion is invariant under the same circumstance (such as the same muscle stiffness, motion speed, and external friction) because the control of the central nervous system keeps the same. Thus the activation level of biceps brachii can be used to predict the angle of elbow joint flexion and extension without considering all involved muscles.

### Hill-Type-Based Muscular Model

In order to calculate the musclotendon force *F*_*B*_, a conventional Hill-type muscular model is adopted [25]. It contains a pair of elements arranged in series: a passive serial element (SE), an active contractile element (CE), and a passive element (PE) arranged in parallel to the previous two. The equations [21, 26] used to calculate the force based on this model are:

4 $$F_{PE,SE} = \left[ {{{F_{\hbox{max} } } \mathord{\left/ {\vphantom {{F_{\hbox{max} } } {e^{s} }}} \right. \kern-0pt} {e^{s} }} - 1} \right]\left[ {e^{{(({s \mathord{\left/ {\vphantom {s {\Delta L_{\hbox{max} } }}} \right. \kern-0pt} {\Delta L_{\hbox{max} } }})\Delta L)}} - 1} \right]$$

5 $$\left\{ \begin{aligned} F_{CE} = F_{\hbox{max} } a(u) \cdot f_{l} \cdot f_{v} \hfill \\ f_{l} = \exp \left( {{ - }0.5\left( {\left( {\Delta L_{CE} /L_{{CE_{0} }} - 0.05} \right)/0.19} \right)^{2} } \right) \hfill \\ f_{v} = 0.1433\left( {0.1074 + \exp \left( { - 1.3\sinh \left( {2.8\frac{{V_{CE} }}{{V_{{CE_{0} }} }} + 1.64} \right)} \right)} \right)^{ - 1} \hfill \\ V_{{CE_{0} }} = 0.5\left( {a(u) + 1} \right)V_{{CE_{\hbox{max} } }} \hfill \\ \end{aligned} \right.$$

6 $$F_{T} = F_{CE} + F_{PE}$$

7 $$a\left( u \right) = \left( {e^{{u(t)R^{ - 1} }} - 1} \right)/\left( {e^{A} - 1} \right)$$

where Δ*L* is the change in length of the element with respect to the slack length, *S* is a shape parameter, *F*_*max*_ is the maximum force exerted by the element for the maximum change in length Δ*L*_*max*_, and *F*_*PE,SE*_ is the passive force generated by the PE or SE depending on the set of parameters used. *F*_*T*_ is the total force exerted by the muscle. *a*(*u*) is the activation level of a muscle.

The SE element presents the force generated by the deformation of the tendon, which is considered to be zero, and thus the SE element is ignored in this study. For the voluntary elbow flexion and extension considered here, the PE element can also be ignored. The muscular force *F*_*B*_ can thus be calculated from Eqs. (5) to (7).

However, accurate estimation of parameters Δ*L*_*CE*_ and *V*_*CE*_ is not easy. According to the musculoskeletal model build in Sect. 2.3 and the assumption that the tendon is stiff, Δ*L*_*CE*_ can be defined as:

8 $$\Delta L_{CE} = l\,\cos \,\theta = \alpha L\cos \,\theta = \alpha L_{{CE_{0} }} \cos \,\theta$$

where *α* is the ratio of *l* to *L*.

*V*_*CE*_ can be defined as:

9 $$V_{CE} = dl\,\cos \,\theta /dt = - \dot{\theta }l\,\sin \,\theta$$

According to a previous study [27], *V*_*CE0*_ can be regarded as 10*L*_*CE0*_/s for the upper limb muscles in most of cases. Given this condition, the following equation is obtained:

10 $$\frac{{V_{CE} }}{{V_{{CE_{0} }} }} = - \frac{{\dot{\theta }\alpha L_{{CE_{0} }} \sin \theta }}{{10L_{{CE_{0} }} }} = - 0.1\alpha \dot{\theta }\,\sin \,\theta$$

Substituting Eqs. (8) and (10) back into the Hill-type model and rewriting the term *F*_*B*_ with the detailed equations yields:

11 $$F_{\hbox{max} } a \times f_{l} (\cos \,\theta ) \times f_{v} \left( {\dot{\theta },\,\sin \,\theta } \right) = C_{1} + A_{1} \ddot{\theta }\,\sin \,\theta$$

Taking the natural logarithm of both sides yields:

12 $$C_{2} + \ln \left( {\exp \left( {uR^{ - 1} } \right) - 1} \right) + \ln \left( {f_{v} \left( {\dot{\theta }\,\sin \,\theta } \right)} \right) = \ln \left( {A_{1} \ddot{\theta }\,\sin \,\theta + c_{1} } \right) + (\alpha \,\cos \,\theta - 0.05)^{2}$$

where *C*_*2*_ and *C*_*3*_ equal ln(*F*_*max*_(exp(*A*) − 1)^−1^) and 0.5 × 0.19^−2^, respectively. The value of term ln*f*_*v*_(*dθ,*sin*θ*) is around zero in this circumstance, and thus ignored. Since the value of *c*_*1*_ is much larger than that of *A*_1_d^*2*^*θsinθ*, the term ln(*A*_1_d^*2*^*θsinθ* + *c*_*1*_) can be simplified as ln*c*_*1*_ and we use *C*_*1*_ to instead for the purpose of keeping mathematic unification. Equation (12) can thus be further simplified as:

13 $$C_{2} + \ln \left( {\exp \left( {uR^{ - 1} } \right) - 1} \right) = C_{1} + C_{3} (\alpha \,\cos \,\theta - 0.05)^{2}$$

where *u* represents the muscle activation level, which can be calculated from EMG signals, and *θ* is the upper limb elbow joint angle.

In Eq. (13), the term ln(exp(*uR*^−1^) − 1) can be represented as a quadratic polynomial with variable *uR*^−1^. Then, Eq. (13) can be transformed into Eq. (14), which has a quadratic-like relationship between cos^2^*θ* and *uR*^−1^. According to the experimental results (discussed in the next section), this quadratic-like relationship is extremely strong during the upper limb elbow flexion period.

14 $$C_{2} + \sum\nolimits_{i = 0}^{2} {a_{i} \left( {uR^{ - 1} } \right)^{i} = C_{1} + C_{3} (\alpha \,\cos \,\theta - 0.05)^{2} }$$

### Muscle Activation Level

The EMG signals can directly reflect the muscle activation level [*a*(*u*)]. Raw EMG signals should be filtered by a high-pass filter to remove any DC offsets or low-frequency noise and then rectified. Sometimes, these rectified signals are directly transformed into muscle activation levels by dividing them by the peak rectified EMG value obtained during the MVC test. Some researchers suggest that a more detailed model of muscle activation dynamics is warranted in order to characterize the time-varying features of the EMG signal. In this paper, a discretized recursive filter is used.

A discretized recursive filter with a continuous form of a second-order differential equation was implemented:

15 $$u(t) = {{Md^{2} e(t)} \mathord{\left/ {\vphantom {{Md^{2} e(t)} {{{d^{2} t + Bde(t)} \mathord{\left/ {\vphantom {{d^{2} t + Bde(t)} {dt + Ke(t)}}} \right. \kern-0pt} {dt + Ke(t)}}}}} \right. \kern-0pt} {{{d^{2} t + Bde(t)} \mathord{\left/ {\vphantom {{d^{2} t + Bde(t)} {dt + Ke(t)}}} \right. \kern-0pt} {dt + Ke(t)}}}}$$

where *M*, *B*, and *K* are the constants that define the dynamics of Eq. (15) and *e*(*t*) is the processed EMG signal. This equation can be expressed in discrete form using backward differences:

16 $$u(t) = \alpha e(t - d) - \beta_{1} u(t - 1) - \beta_{2} u(t - 2)$$

where *d* is the electromechanical delay and *α*, *β*_1_, and *β*_2_ are the coefficients that define the second-order dynamics. Selection of the values for *α*, *β*_1_, and *β*_2_ should follow the following restrictions:

17 $$\beta_{1} = \gamma_{1} + \gamma_{2}$$

18 $$\beta_{1} = \gamma_{1} \times \gamma_{2}$$

19 $$\left| {\gamma_{1} } \right| < 1$$

20 $$\left| {\gamma_{2} } \right| < 1$$

21 $$\alpha - \beta_{1} - \beta_{2} = 1$$

in order to guarantee the stability of the equation and that neural activation does not exceed 1.

The calculation results should be filtered by a low-pass filter (with a cut-off frequency of 3–10 Hz) because the muscle naturally acts as a filter, resulting in that force changing frequency is much lower than amplitude changing frequency of EMG signals. Here, the cut-off frequency was set even lower, to around 0.5–1 Hz. As a consequence, some energy of the original signals was filtered, but a more smooth muscle activation level curve was obtained. Since the purpose of this paper is to represent the elbow joint angle, not clinical analysis, this processing is reasonable.

### State Switching Model

**Fig. 3:**
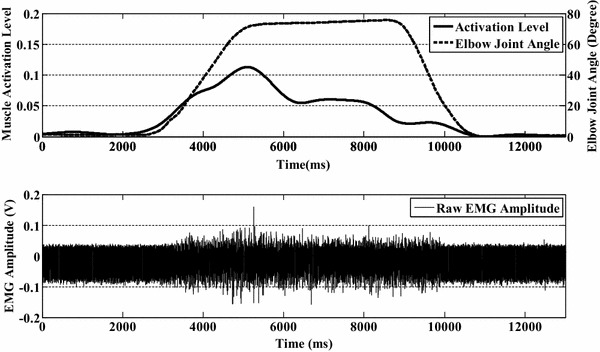
One set of experimental results from one subject obtained during forearm flexion and extension. In the *upper figure*, the *solid line* is the normalized muscle activation level estimated from EMG signals and the *dashed line* is the upper limb elbow joint angle. The *lower figure* shows the raw EMG signals recorded from the biceps brachii

**Fig. 4:**
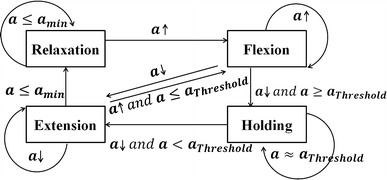
Proposed state switching model. The four states are relaxation, flexion, holding, and extension. The switching thresholds for each state are determined in the calibration process

Although the relationship between EMG signals (muscle activation level) and elbow joint angle seems simple from Eq. (13), the actual relation is more complicated. Figure 4 shows one set of experimental results of normalized muscle activation level during the motion of elbow flexion and extension. There are four periods: relaxation period, elbow flexion period (part A in Fig. 3), holding period (part B in Fig. 3), and elbow extension period (part C in Fig. 3). In the flexion period, the activation level has a quadratic-like relation with the elbow joint angle, which corresponds to Eq. (14). In this period, the type of muscle contraction is concentric contraction. The interesting part is the connection portion between the flexion period and the holding period, where the musclotendon force decreases rapidly and then plateaus, which is similar to overshoot in control theory. This result can be explained by Eq. (2). During the motion of elbow flexion, the force or torque exerted by the muscle can be represented as:

2 $$F_{B} l\sin \,\theta = mg\sin \,\theta L + I\ddot{\theta }$$

When the forearm is held at 90° from the upper arm, the desired torque can be represented as:

22 $$F_{B} l = mgL,(\theta = 90^{ \circ } )$$

Compared with Eq. (22), there is an extra acceleration term in Eq. (2), resulting in a higher force level in the elbow flexion period. As the muscle activation level directly reflects the musclotendon force, the activation level is higher in the flexion period than in the holding period. Another reason is the transformation of muscle contraction type from concentric to isometric. However, this issue is beyond the scope of this paper. During the period of elbow extension, the muscle activation level decreases with decreasing elbow joint angle. Furthermore, during the extension period, the muscle contraction type changes to active shortening.

Given this situation, a state switching model (as shown in Fig. 4) is developed for elbow joint angle prediction. The input of this switching model is the muscle activation level. There are four states in this state switching model, namely relaxation, flexion, holding and extension states, which correspond to the elbow joint motions with the same names. The relaxation state is the initial state, at which the forearm is 180° from the upper arm in the sagittal plane. When the muscle activation level increases, the state changes to the flexion state. The flexion state changes to the holding state only when the activation level stops increasing and the value exceeds a threshold. When the level decreases, the state changes to the extension state. In the holding state, the state can only change to the extension state when the activation level starts to decrease. The extension state changes to the relaxation state when the value of the activation level decreases to an inactivation level, and then changes to the flexion state when the force value increases.

Although a low-pass filter (with a cut-off frequency of 0.5–1 Hz) was used when transforming EMG signals into muscle activation levels, a further simple value rectification filter with an empirical change range of 1–3 % was used to stabilize the activation level in the relaxation state.

Another problem in the state switching model is the discontinuity of the representation elbow joint angle value between the flexion state and the extension state, especially when the state changes from the holding state to the extension state. It is assumed that this is caused by the acceleration term in Eq. (2). The force exerted at the end of the flexion state is higher than that at the beginning of the extension state even though the elbow joint angle is almost the same. Because a continuous quadratic-like function is implemented, the outputs of this function between the two states are discontinuous. In this paper, a simple proportional gain is used to solve this problem:

23 $$p = \frac{{\theta_{\hbox{max} } }}{{\theta{\prime}_{\hbox{max} } }}$$

where *θ*_max_ is the elbow joint angle at the end of the flexion state and *θ′*_max_ is the elbow joint angle at the beginning of the extension state. According to the definitions of flexion and extension states, the last value at the end of the flexion state is the maximum elbow joint angle at the flexion state and the first value at the beginning of the extension state is the maximum elbow joint angle at the extension state.

### Schematic of Designed Elbow Joint Continuous Representation Method

In the pre-processing step, raw EMG signals are filtered by a high-pass fourth-order Butterworth filter with a cut-off frequency of 10 Hz and then are processed using Eq. (16) to get the feature estimation of *u*(*t*). After *u*(*t*) is filtered with a low-pass second-order Butterworth filter (with 0.5–1 Hz cut-off frequency), the results are used as the input of Eq. (7). The outputs are the muscle activation level (*a*). Then, the activation level is rectified by 1–3 % to reduce the drifting effect caused by the characteristics of EMG signals. The rectified activation level is used as the input to the proposed state switching model to obtain a representation of the elbow joint angle, which is used as the control reference input for the exoskeleton device. The proposed Hill-type-based muscular model is combined in the state switching method.

## Results and Discussion

A continuous upper limb elbow flexion and extension experiment and a consecutive stepping experiment were conducted to estimate the efficiency of the proposed method. These experiments were conducted on ten subjects for 4 days. Each subject performed the experiment ten times each day.

### Evaluation of Proposed Musculoskeletal Model

**Fig. 5:**
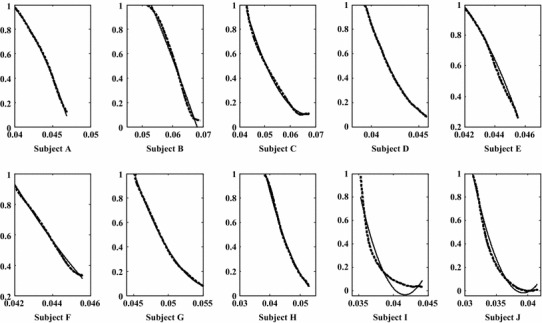
One set of experimental results of simplified musculotendon prediction results obtained using only EMG. The *solid blue line* is the simplified prediction results and the *green dashed line* is the prediction results with EMG and MTx sensor

To evaluate the proposed musculoskeletal model, all recorded data from the ten subjects during the 4 days were fitted using the curve fitting tools of MATLAB with quadratic polynomial equations. The inputs were values of the muscle activation level during the flexion motion and the outputs were values of cos^2^θ. Some bad data caused by electrodes sliding on the skin surface were ignored. Figure 5 shows one set of model evaluation results from the ten subjects. The dashed lines are the results calculated with data recorded from EMG electrodes (to get the muscle activation level) and from the MTx sensor (to get elbow joint angles). The solid lines are prediction results based on the proposed model. Table 1 lists detailed information (mean ± SD). The experimental results show that the average values of the correlation coefficient is above 0.97 for all ten subjects. Although a linear relationship between muscle activation level and cos^2^θ was found for some subjects (in Fig. 5, subjects B and F have correlation coefficients of 0.95 and 0.94, respectively), the quadratic-like relationship has a higher correlation coefficient (with correlation coefficients of 0.97 and 0.98) than that of the linear one in the same case. In other cases (in Fig. 5, subjects I and J), the quadratic-like relationship is more suitable (linear relationship has correlation coefficients of 0.86 and 0.85 and quadratic-like one has 0.97 and 0.98).

**Table 1:** Correlation coefficients between experimental data and proposed model

Day	Subject									
	A	B	C	D	E	F	G	H	I	J
1	0.99 ± 0.01	0.99 ± 0.01	0.98 ± 0.01	0.98 ± 0.01	0.99 ± 0.00	0.98 ± 0.01	0.98 ± 0.01	0.98 ± 0.01	0.97 ± 0.02	0.98 ± 0.01
2	0.98 ± 0.01	0.98 ± 0.01	0.98 ± 0.01	0.98 ± 0.01	0.98 ± 0.01	0.98 ± 0.01	0.98 ± 0.02	0.99 ± 0.01	0.97 ± 0.01	0.97 ± 0.01
3	0.99 ± 0.01	0.97 ± 0.02	0.97 ± 0.02	0.99 ± 0.01	0.97 ± 0.02	0.97 ± 0.01	0.99 ± 0.01	0.97 ± 0.02	0.97 ± 0.01	0.98 ± 0.01
4	0.98 ± 0.01	0.99 ± 0.01	0.97 ± 0.01	0.99 ± 0.01	0.99 ± 0.01	0.98 ± 0.01	0.98 ± 0.01	0.98 ± 0.02	0.97 ± 0.01	0.97 ± 0.01

The range of the upper limb motion was constrained from 0° to 90°. The change of muscle length can thus be represented by the cosine function without considering the phase change of *θ*. This range avoids the problem of EMG misdetection introduced by the sliding of electrodes on the skin surface. The subjects were asked to move their forearms with angular velocity (*dθ*) of 30°/s during the entire experiment. This was guaranteed by subjects’ pre-practice. However, the angular velocity is not necessary to be strictly constrained. In Eq. (12), *dθ*, which is ln[*f*_*v*_(*dθ,sinθ*)], can be considered as zero because the value of *f*_*v*_(*dθ,sinθ*) is around 1 (0°/s < *dθ* < 90°/s). *A*_1_d^*2*^*θsinθ* + *c*_*1*_ can be simplified as *c*_*1*_ because *c*_*1*>>_*A*_1_d^*2*^*θsinθ* if d^*2*^*θ* is not very large. As a consequence, the angular velocity of the forearm is not needed to be strictly constrained.

### Continuous Elbow Joint Angle Prediction

**Fig. 6:**
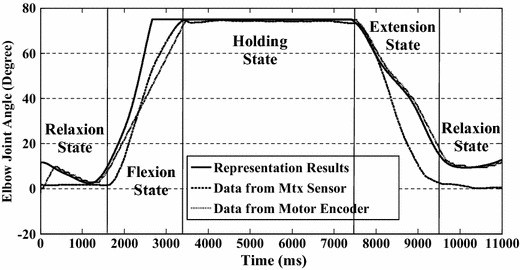
One set of representation results with proposed method from one subject. The *solid line* is the represented results and the *dashed line* is the recorded results from MTx sensor. The trajectory of the exoskeleton device is plotted with a *dotted line*, which is good agreement with the prediction results

Figure 6 shows one set of the elbow joint angle prediction results obtained using the proposed method. The calculated elbow joint angles are plotted with a solid line and the recorded elbow joint angles obtained using the MTx sensor are plotted with a dashed line. Trajectory of the exoskeleton device is plotted with a dotted line. The different states are divided using black lines. In state 1 (relaxation state), the prediction results and recorded results are all zero. Actually, small changes in the muscle activation level can be observed in this period due to the small changes in EMG signals. These small changes may make the current state change to the next state and cause errors. A rectification method is thus implemented to stabilize the changes. In state 2 (flexion state), there is usually a time lag (about 100–200 ms) at the end of this state between the recorded data and prediction results. This time lag is caused by the transition from the flexion state to the holding state. The flexion state changes to holding state when the input (the muscle activation level) for the state switching exceeds a threshold, which is pre-determined. However the real desired threshold changes with the variation of EMG signals. As a consequence, the constant pre-determined threshold makes the prediction of holding state backwardly. In state 3 (holding state), the elbow joint remains in a certain position (75° in this case). When the state changes to the extension state, the values of prediction results decrease with decreasing muscle activation level. The correlation coefficients and root-mean-square (RMS) errors between prediction results and recorded ones of the ten subjects are listed in Table 2.

**Table 2:** (a) Correlation coefficients and (b) RMS errors (degrees) between prediction results and recorded results

Day	Subject									
	A	B	C	D	E	F	G	H	I	J
A										
1	0.96 ± 0.02	0.91 ± 0.01	0.94 ± 0.03	0.95 ± 0.01	0.98 ± 0.02	0.96 ± 0.01	0.96 ± 0.07	0.97 ± 0.02	0.94 ± 0.09	0.93 ± 0.05
2	0.98 ± 0.01	0.92 ± 0.05	0.94 ± 0.05	0.95 ± 0.04	0.95 ± 0.04	0.96 ± 0.03	0.98 ± 0.04	0.97 ± 0.03	0.97 ± 0.01	0.97 ± 0.03
3	0.98 ± 0.04	0.91 ± 0.09	0.93 ± 0.07	0.93 ± 0.06	0.95 ± 0.05	0.91 ± 0.08	0.94 ± 0.06	0.97 ± 0.02	0.94 ± 0.01	0.92 ± 0.01
4	0.94 ± 0.06	0.95 ± 0.04	0.97 ± 0.01	0.95 ± 0.07	0.94 ± 0.06	0.97 ± 0.01	0.92 ± 0.08	0.95 ± 0.05	0.95 ± 0.01	0.96 ± 0.02
B										
1	9.78 ± 3.79	8.10 ± 2.59	5.20 ± 2.80	9.22 ± 2.12	5.60 ± 0.34	9.17 ± 2.80	9.47 ± 1.37	9.70 ± 0.13	8.64 ± 1.60	5.33 ± 1.27
2	7.33 ± 2.14	7.56 ± 2.31	4.32 ± 2.21	7.31 ± 3.31	5.55 ± 1.21	7.78 ± 2.57	8.33 ± 2.12	9.05 ± 2.11	5.31 ± 2.21	4.21 ± 3.17
3	6.23 ± 4.11	7.71 ± 2.77	5.78 ± 3.33	8.21 ± 2.23	6.04 ± 2.11	7.31 ± 2.23	7.78 ± 3.16	7.73 ± 3.01	7.04 ± 2.13	5.78 ± 2.00
4	7.35 ± 2.11	8.78 ± 2.11	4.32 ± 2.45	5.45 ± 3.13	5.57 ± 3.14	5.32 ± 2.85	9.01 ± 2.11	7.00 ± 1.31	6.66 ± 3.01	6.05 ± 1.15

The proposed state switching model mitigates the effect of the acceleration term in Eq. (2) and the transitory changes between different types of muscle contraction. In the beginning, a general quadratic-like relationship was developed [as shown in Eq. (13)] to map the muscle activation level to the elbow joint angle. However, the overshoot (as shown in Fig. 4) made it very difficult or impossible to obtain accurate representations using only one function. As for the purpose of using only EMG signals, the measurement of acceleration is out of consideration. Given this situation, different states were used to express the different parts and a connection was made between them. Although the EMG signals are non-stationary, the motion of the upper limb is continuous, i.e., there is no jump point between two continuous elbow joint angles. The changes of EMG signals or muscle activation levels follow a continuous trend during motion. In the experiment, a strong quadratic-like relationship between the calculated muscle activation level and the elbow joint angle during the motion of flexion was found. This relation well corresponds to the proposed Hill-type-based muscular model. Thus, a quadratic mapping function was developed based on the data from the motion of the subject performing upper limb flexion and extension voluntarily (the angular velocity was constrained). When the motion changes from flexion to holding, the contraction motion of the muscle changes from concentric to isometric and the effect of acceleration disappears. The muscle activation level usually drops to a relatively low level. A single quadratic function directly reflects this kind of drop while the actual position of the forearm remains unchanged. Thus, two states are used to divide these two parts (flexion and holding). When the subject goes on extending their upper limb, a jump point appears if the same mapping function is directly used. Thus, a proportional gain is implemented to smooth this jump point. The extension state is therefore used to denote this part.

**Fig. 7:**
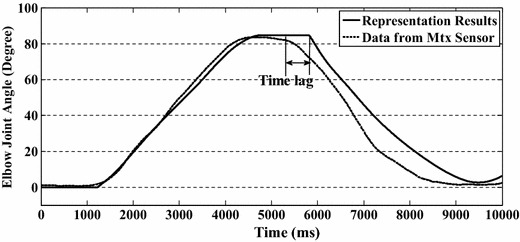
Prediction results of flexion and extension motion without holding motion. The proposed state switching method gives rise to distortion or time lag around the peak point of the motion

Nevertheless, the proposed state switching model may give rise to distortion or time lag in some cases. In Fig. 7, the motion is forearm flexion and then extension, without a holding period during flexion and extension. There is a time lag between the flexion and extension in the prediction results. This is because the state changes from flexion to holding and then to extension. It takes some time (as long as the time lag) for the model to change state from holding to extension. This time lag depends on the decreasing rate of EMG signals (*γ*), the difference between peak muscle activation levels (*a*_*P*_), the threshold set for the holding state, and a range value (*a*_*r*_: 1–3 %) that is used to reduce the influence of the non-stationarity of EMG signals. The time lag can be defined as:

24 $$t_{lag} = \frac{{F_{p} - F_{t} (1 - a_{r} )}}{\gamma }$$

where the only parameter which can be controlled is *a*_*r*_. However the influence of *a*_*r*_ is much less than that of the other parameters. Thus, *t*_*lag*_ can be regarded as an inherent defect of this model caused by the non-stationarity of EMG signals. Although this time lag appears in certain circumstances, it does not affect all results, i.e. this lag, does not accumulate in the state switching method.

### Consecutive Stepping Elbow Joint Angle Prediction

**Fig. 8:**
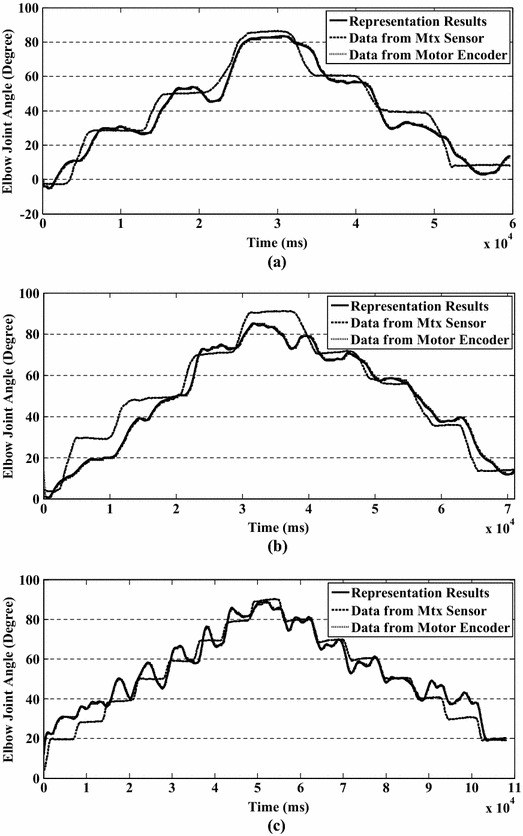
Consecutive stepping test results for increment angles of **a** 30°, **b** 20°, **c** 10°

To evaluate the proposed method in a more complicated circumstance, a consecutive stepping test was performed by five of the ten subjects. One set of the experimental results is shown in Fig. 8 and the detailed information for the five subjects is given in Table 3. In the stepping experiment, the subjects were asked to perform the movement with an angular velocity of 30°/s. The experimental results show that the RMS errors between prediction results and recorded ones increase with decreasing increment angle. For the 30° increment angle case, the prediction results corresponded to the recorded data well and only small errors were found (with mean RMS error of 5.67°). In this case, the ULED can follow the motions of the active upper limb quickly and no obvious deviation was felt by subjects. In the 20° increment angle case, relatively large deviations or floats from the recorded elbow joint angles were observed in the data (with mean RMS error of 8.02°) and reported by subjects. Although the ULED can follow the motions of the subject’s active upper limb, some trembling was sensed. For the 10° increment angle case, the deviations between prediction results and recorded ones were large (with mean RMS error of 12.99°). Only a general trend can be found from the data and obvious trembling was sensed by subjects.

**Table 3:** RMS errors between prediction results and recorded results in consecutive stepping test

Increment	Subject				
	A	C	E	G	I
30	4.40 ± 3.15	5.40 ± 2.21	6.51 ± 3.11	5.32 ± 4.21	6.71 ± 4.00
20	6.61 ± 3.71	8.83 ± 4.94	7.139 ± 3.90	9.21 ± 2.11	8.31 ± 3.57
10	15.40 ± 3.15	17.40 ± 3.12	17.35 ± 4.12	19.35 ± 3.15	17.44 ± 4.23

The experimental results show that the efficiency of the proposed method decreases with decreasing of increment angle. According to the experimental results, the proposed method provides a “good, faire, and poor” predictions of elbow joint angle with increment angles of 30°, 20°, and 10°, respectively. One of the reasons for that the efficiency of the proposed method decreases with decreasing of increment angle is that the trend of EMG signals tends to become more unstable or the amplitude of ripple of EMG signals tends to become wider with decreasing increment angle. The wide ripple of EMG signals directly influences the calculation of muscle activation level, i.e., there are ripples in muscle activation levels. The activation levels thus become unstable as well. This phenomenon was found for all five subjects during the consecutive stepping test. But this kind of phenomenon doesn’t appear in the continuous motion test. This phenomenon indicates that the subject must provide more effort to achieve the task in the consecutive stepping text than in the continuous motion text and the fluctuation in EMG signals during consecutive stepping test reflects upon this effort. According to the experimental results, the proposed method can provide suitable predictions within increments of 20°–30°.

## Conclusion

This paper proposed an upper limb elbow joint representation method that uses only single-channel EMG signals. EMG signals are recorded from the biceps muscle and a discretized recursive filter is implemented to calculate the muscle activation level from these signals. A modified Hill-type muscular model was implemented to build a quantitative relationship between the elbow joint angle and the muscle activation level (or EMG signals). After some simplification, a quadratic-like relationship was developed. The experimental results indicate that the proposed model works well during flexion and extension motion. Furthermore, a state switching model was developed to avoid the influence of acceleration and transit between muscle contraction states. A consecutive stepping experiment was conducted to test the feasibility of the proposed method with more free motion. Experimental results indicate that this method can provide suitable prediction results with RMS errors of below 10° in continuous motion and RMS errors of below 10° in stepping motion with 20° and 30° increments. The proposed method can be implemented for real-time calculation, human–machine interaction and may be useful for neurorehabilitation. The proposed method will be implemented for a bilateral rehabilitation exercise in the future.
